# The microendemic *Aegla expansa* (Aeglidae) survives in highly disturbed micro-basins of southern Chile

**DOI:** 10.3897/zookeys.1268.167269

**Published:** 2026-02-04

**Authors:** Andrés Muñoz-Pedreros, Heraldo V. Norambuena, Carlos G. Jara, Pedro Jara-Seguel, Marcela Guerrero, Marilyn González-Urrutia

**Affiliations:** 1 Núcleo de Estudios Ambientales (NEA), Departamento de Ciencias Ambientales, Facultad de Recursos Naturales, Universidad Católica de Temuco, Temuco, Chile Universidad Austral de Chile Valdivia Chile; 2 Centro de Estudios Agrarios y Ambientales (CEA), Valdivia, Chile Universidad de La Frontera Temuco Chile; 3 Centro Bahía Lomas, Facultad de Ciencias, Universidad Santo Tomás, Concepción, Chile Universidad Católica de Temuco Temuco Chile; 4 Universidad Austral de Chile, Valdivia, Chile Centro de Estudios Agrarios y Ambientales (CEA) Valdivia Chile; 5 Facultad de Agronomía y Ciencias Forestales, Universidad de La Frontera, Temuco, Chile Universidad Santo Tomás Concepción Chile

**Keywords:** *
Aegla
*, COI, endangered species, freshwater crabs, Hualqui, phylogeny

## Abstract

*Aegla expansa* was described from an exoskeleton by Jara (1992). The type specimen (holotype) of the species was obtained from the hamlet of La Leonera, approximately six km east of the town of Hualqui in the Biobío region of Chile. Since this find, no further biological information has been generated on this species, and it was even thought to have become Extinct in the Wild. By systematic sampling in the commune (district) of Hualqui, we were able to find populations of *A. expansa* in many of the district’s rivers. In this work, we present the first biological information on the species and assess its phylogenetic position within the genus *Aegla*.

## Introduction

Freshwater crabs of the Aeglidae family are restricted to southern South America (Chile, Brazil, Bolivia, Uruguay, Paraguay, and Argentina). They all belong to the genus *Aegla*, which contains 94 species (Mollmann et al. 2024; WoRMs Editorial Board 2025). In Chile, 18 species of malacostracan crustaceans inhabit freshwater wetlands along river and lake shores, with a high degree of endemism (Manning and Hobbs 1977; Bond-Buckup and Buckup 1994; Jara et al. 2006). Their archaic zoogeographic relationships are thought to be Gondwanan (Bond-Buckup et al. 2008; Xu et al. 2009; Bartholomei-Santos et al. 2019; Zimmermann et al. 2019).

The species *Aegla expansa* Jara, 1992 was described from an exoskeleton by Jara (1992). The type specimen (holotype) of the species was obtained from the area of La Leonera, approximately six km east of the town of Hualqui in the Biobío region of southern Chile (36°58'38"S, 72°52'04"W). The diagnostic characteristics of this species are: large chelae with a very expanded subdisciform palmar crest; posterior end of the palmar crest shaped like an earlobe, the edge of which fits into a wide sinus on the distal dorsal end of the carpus; rough surface of the chelae; face broad at the base, ligulate, excavated on both sides of the facial carina; wide extraorbital sinus, separated from the orbital by two superimposed spines; gastric and cardial areas markedly convex and protuberant; serrated branchial margins; smooth, flat ventral face of 4^th^ thoracic sternum; and acute anterolateral angle of the second abdominal segment (Jara 1996).

In a working meeting of specialists in native Chilean decapods, its state of conservation was classified as ‘Data Deficient’ due to the lack of information (Bahamonde et al. 1998). In an excursion to the *terra typica* in February 2000, only two juveniles (3 mm CL) of uncertain affiliation were collected (after two hours of capture effort, Jara 2005). The streambed was found to be practically destroyed by human activities, including exotic forest plantations, road construction, and pollution of water bodies (Jara pers. comm.). The species was classified as ‘Extinct in the Wild’ by Pérez-Losada et al. (2002), and five years later, the holotype and the two unclassified juveniles were destroyed by a fire at the Science Faculty of Universidad Austral de Chile in Valdivia, which consumed the largest existing collection of Chilean aeglids.

No further specimens were collected until 2012, when an expedition of the Centre for Agrarian and Environmental Studies (CEA) collected specimens of *A. expansa* in streams in the commune of Hualqui. As a result, in 2014, the Council of Ministers for Sustainability of Chile (Agreement 6/2014) cancelled the extinct status and classified the species as Endangered (EN B1ab(iii)+2ab(iii)), its current legal status in Chile.

The aims of this study were to: (a) identify new locations where *A. expansa* occurs, (b) characterize the habitats in which this species is found, and (c) propose conservation measures for this critically endangered species. Specifically, the objectives included detecting new distribution sites of *A. expansa* in watercourses near its type locality, and validating its taxonomic identification by evaluating its phylogenetic relationship with other species within the genus *Aegla*.

## Material and methods

### Study area

**Figure 1.**
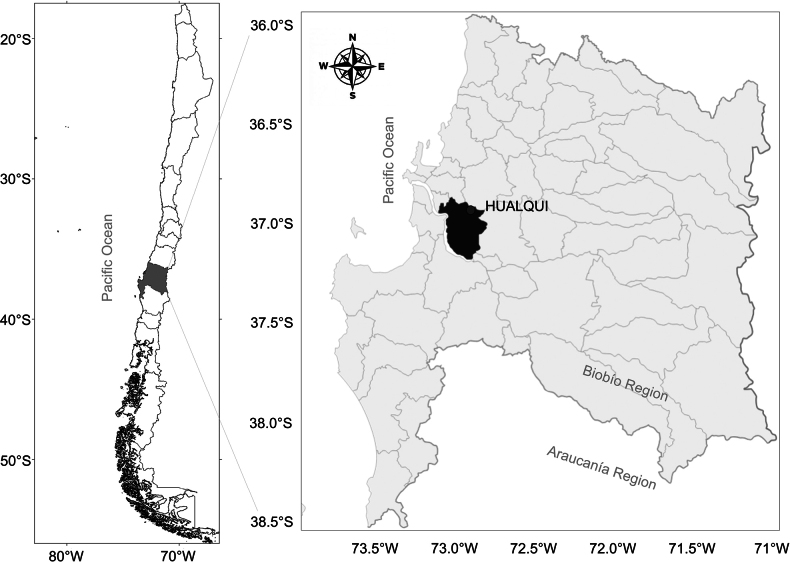
Study area in the commune of Hualqui, southern Chile.

The study area was the commune of Hualqui, 37°00'S to 37°15'S and 72°45'W to 73°00'W, with an area of 530.5 km^2^ (INE 2002) (Fig. 1). The Biobío River dominates the hydrography; it is fed by two micro-basins: the Quilacoya River and the Hualqui Stream, which form sub-basins. Other streams are: La Araucana, San Onofre, Agua Larga, Colliguay, Chanco, and Leonera. The climate is warm temperate with a wet season of seven to eight months and a short dry season in summer. The mean annual temperature is 13 °C, with January the hottest month (17.7 °C) and July the coldest (9 °C). Mean annual precipitation is 1428 mm (PLADECO 2016).

The sites where specimens of the genus *Aegla* were collected were: La Araucana Stream in Hualqui (36°58'09"S, 72°56'40"W); La Araucana Stream in La Leonera (36°56'36"S, 72°55'41"W); Agua Corta Stream (36°58'45"S, 72°53'06"W); Lircay Stream in Millahue (36°59'34"S, 72°49'37"W); Ainahue Stream in Agua Larga (36°58'43"S, 72°52'41"W); San Onofre Stream (37°00'26"S, 72°54'37"W); Fundo El Relajo Stream (37°00'55"S, 72°54'35"W); Chillancito Stream (37°03'50"S, 72°53'13"W); Quilacoya River in Vegas de Diuca (37°04'01"S, 72°52'47"W); Quilacoya river mouth (37°03'02"S, 72°57'45"W); and Biobío River in Quilacoya (37°03'01"S, 72°57'47"W).

### Capture of specimens

The specimens were captured in two periods: 7 and 8 December 2012 and 21–23 December 2015, in water bodies in the study area using a fine-mesh hand net (~5 mm) of 1 m^2^. Each sweep of the net along the bottom was 2 m in length. This was multiplied by the lower edge of the net mouth (1 m) to calculate the sweep area (2 m^2^). From this calculation of the capture effort, we could estimate animal density, i.e., the number of aeglids captured per unit area. The specimens collected were fixed in pure alcohol, identified, and deposited in the decapod crustacean collection of the CEA.

### DNA extraction, amplification and sequencing

To validate the identification of *A. expansa* and to evaluate its phylogenetic position relative to other species of the genus *Aegla*, we obtained mitochondrial COI gene sequences. Genomic DNA was extracted from samples following the protocol of Fetzner (1999), and using the QIAGEN DNeasy kit. We sequenced the mitochondrial COI gene using the primer sequences L5215 and H6313, following the protocol described in (Sorenson et al. 1999). PCR products were sequenced in both directions by automatic sequencing using Macrogen’s ABI3730XL (Seoul, Korea). Sequences were edited using Codon Code Aligner v. 3.0.3 (Codon Code Corporation 2007) and translated into amino acids to corroborate the absence of stop codons. Sequence alignment was conducted in MUSCLE (Edgar 2004), producing a final alignment length of 963 bp for 73 samples (including two samples of *Aegla expansa*). A saturation test was conducted in DAMBE v. 5.2 (Xia et al. 2003) to evaluate the utility of the sequences for phylogenetic analysis. The proportion of invariable sites, a key parameter for the saturation test, was obtained with jModeltest (Darriba et al. 2012). All sequences were deposited in GenBank (Suppl. material 1).

### Phylogenetic analysis

We used the Bayesian inference (BI) approach for phylogenetic reconstruction. We conducted Bayesian analyses using the BEAST v. 1.10.4 programme (Drummond et al. 2012), using ‘coalescent: constant size’for the tree prior, which is suitable for analyses at relatively shallow phylogenetic levels (Drummond et al. 2012). We identified the best-fit nucleotide substitution model using jModeltest 2 (Darriba et al. 2012), which indicated HKY+G+Γ as the best-fit model for COI. We ran all analyses for 100 million generations, sampling every 1000 steps; the first 25% of the data was discarded as burn-in. The convergence of the MCMC analysis was visually examined in TRACER v. 1.6 (Rambaut and Drummond 2009). We analysed posterior output in TRACER v. 1.6 and specified a burn-in of 25%. We used sequences from *Munida subrugosa* (White, 1847), *Munida sanctipauli* Henderson, 1885, *Munida microphthalma* Milne-Edwards, 1880, *Munida iris* Milne-Edwards, 1880, and *Munida zebra* (Macpherson, 1994) as the outgroup.

## Results and discussion

### Phylogenetic position

**Figure 2.**
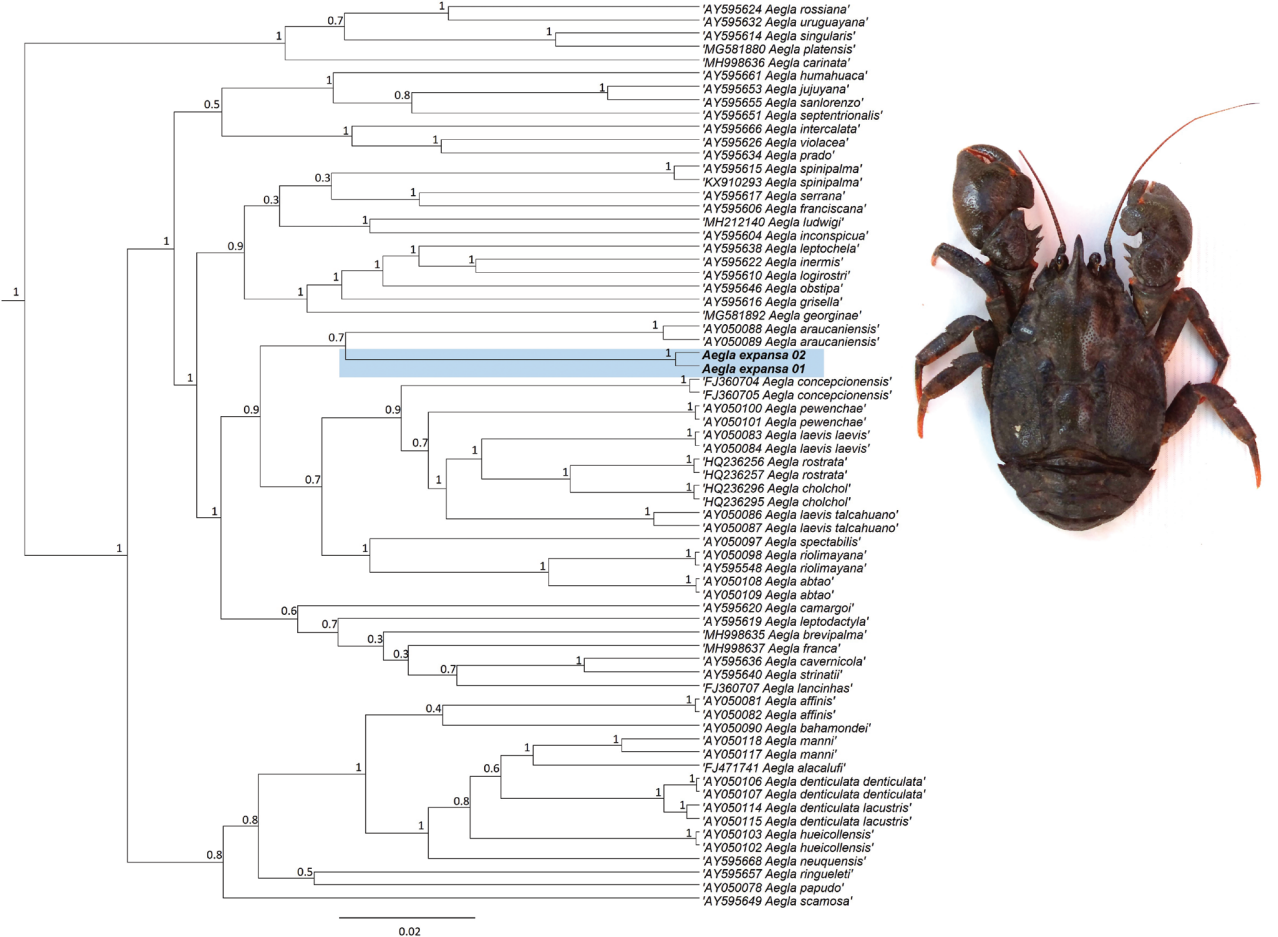
Bayesian molecular phylogeny based on mitochondrial COI gene. Values on nodes represent posterior probability values (PP). The figure depicts the phylogenetic position of *Aegla expansa* relative to other species within the genus, including those occurring outside Chile, shown in light blue. Inset photography of *Aegla expansa*. Outgroups not shown.

Sequences of 964 bp in length for the COI locus were obtained. The BI trees based on COI sequences inferred *Aegla* as a monophyletic group (posterior probability PP of 1.0; Fig. 2). *Aegla expansa* was identified as a sister species of *A. araucaniensis* Jara (1980) (PP 0.7) and both species are sisters of a clade that includes mostly Chilean species as *A. concepcionensis* Schmitt, 1942, *A. laevis* (Latreille, 1818), *A. cholchol* Jara & Palacios, 1999, *A. rostrata* Jara, 1977, *A. pewenchae* Jara, 1994, *A. spectabilis* Jara, 1986, *A. riolomayina* Schmitt, 1942 and *A. abtao* Schmitt, 1942 (Fig. 2). The taxon *A. expansa* is separated from the other *Aegla* species by a genetic distance of at least 4% (albeit based on one gene).

### Records of *A. expansa* in new locations

**Figure 3.**
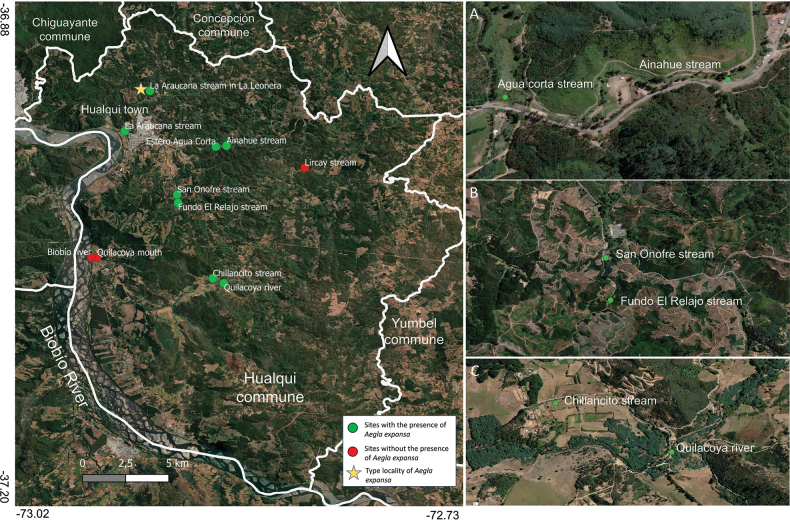
Sampling sites with and without the presence of *Aegla expansa* in the commune of Hualqui, southern Chile. **A**. Close-up of sites ‘Estero Agua Corta’ and ‘Arroyo Ainahue’; **B**. Close-up of sites ‘Estero San Onofre’ and ‘Estero fundo El Relajo’; **C**. Close-up of sites ‘Arroyo Chillancito’ and ‘Río Quilacoya’.

**Figure 4.**
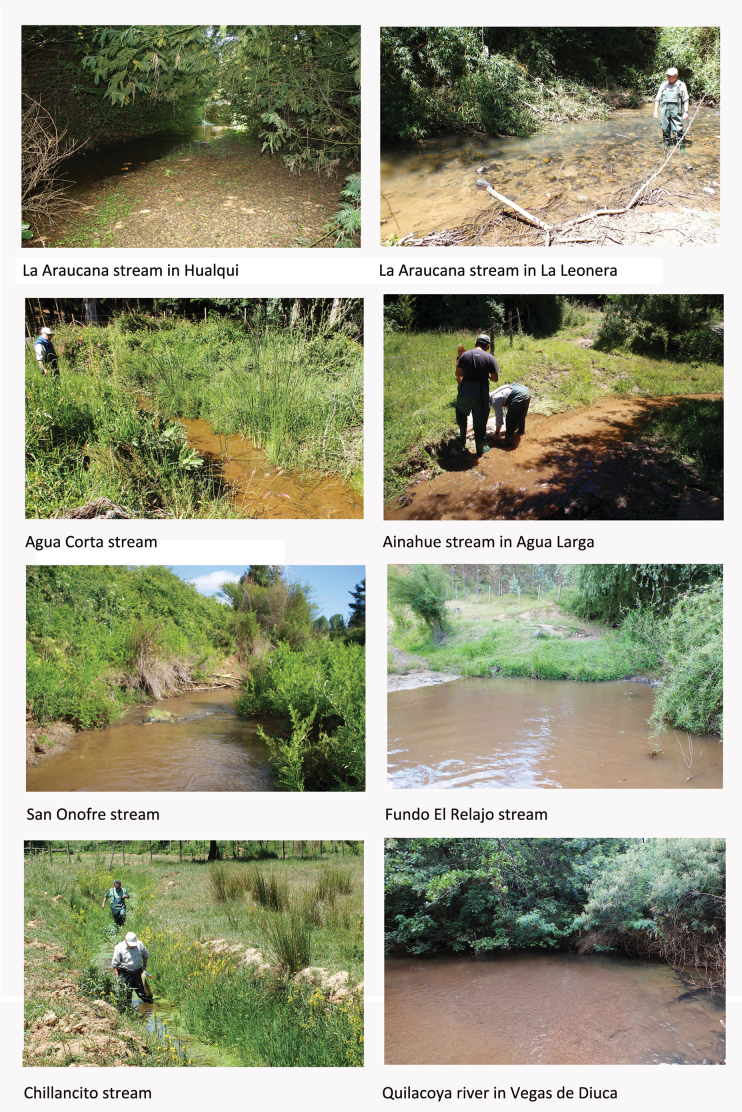
Sites with the presence of *Aegla expansa* in the commune of Hualqui, southern Chile.

New records of *Aegla expansa* were obtained in eight water bodies in the commune of Hualqui. The mean density in spring 2012 was calculated as 26 specimens per 100 m^2^ (seven rivers and streams), with values ranging from 5–89 ind. per 100 m^2^. The physical and chemical parameters in the streams varied as follows: dissolved oxygen (ppm) between 8.72 and 10.75; temperature (°C) between 17.1 and 19.9; pH (units) between 7.1 and 7.5; and conductivity (μS cm-1) between 0.04 and 0.06. The new sites for the species are described below (Figs 3, 4).

1. La Araucana Stream in Hualqui and close to its confluence with the Biobío River (30 m a.s.l.). This is a small, stony river with arboreal vegetation, mainly exotic, along the banks. In eleven sweeps, we collected one specimen of *A. expansa*. There is a patch of rubble and large, round stones, with a lot of rubbish, where the current is turbulent and forms a waterfall. However, no specimens were recorded there. Upstream of the bridge, the bottom is flat and smooth, consisting of sand and quartziferous mica gravel, interrupted by some half-buried pieces of trunks or sawn logs, under which we recorded eight specimens of *Aegla araucaniensis*.
2. La Araucana stream at La Leonera, in Fundo El Molino (21 m a.s.l.), east of the town of Hualqui. This site is a stream with clasts of different sizes. In nine sweeps we collected nine specimens of *A. expansa* in a patch of coarse gravel in full sun, with yellowish-grey, semi-turbid water over stones with a layer of filamentous fungi (apparently due to contamination with domestic sewage). According to the coordinates, this is the site where the holotype of the species was collected, which was lost in the fire at the Science Faculty of Universidad Austral de Chile.
3. Agua Corta Stream at the San Luis bridge, 2 km from Hualqui (69 m a.s.l.). This is a stream less than 1 m wide and 45 cm deep, with a sandy bottom and groups of clasts, in a small, damp valley. It is flanked by reeds and has dense aquatic vegetation, with crops on either side (e.g., potatoes *Solanum tuberosum* L.). A paved road runs along the stream, and it has little bankside vegetation apart from some isolated clumps of scrub. In ten sweeps we collected one juvenile specimen of *A. expansa*. Little rubbish was recorded around the site.
4. Lircay Stream at Millahue, Fundo El Nerco between Vaquería and Huidanqui (99 m a.s.l.). This is a tributary of the Quilacoya River with gravel and small to medium-sized round stones, dense bankside vegetation of mixed scrub. In eight sweeps, no specimens of *A. expansa* were collected, but eight specimens of *A. concepcionensis* were recovered.
5. Ainahue Stream at Agua Larga, at the entrance to Diucalemu farm, 300 m up the valley from the bridge (86 m a.s.l.). It is a small stream with round stones and deep pools in a sandy bottom with scattered branches. In two sweeps, we collected two specimens of *A. expansa*. There is no rubbish around the site.
6. San Onofre Stream, at the Puente Negro bridge (4 m a.s.l.); this is a medium-sized stream with scrubby bankside vegetation and a sand and coarse gravel bottom, interrupted by rapids and deep pools. The water is acceptably transparent, although it contains dissolved and fine particulate organic matter. In 19 sweeps, we collected 19 specimens of *A. expansa*. The local threats to the species are severe human disturbance and plentiful rubbish on the banks and in the stream bed.
7. Fundo El Relajo Stream, between Puente Negro bridge and Santa de Piedra (24 m a.s.l.). The stream has rapids, a bottom of rounded stones, and deeper pools with some mud; the water is semi-turbid in a narrow, deep bed, with cattle pastures nearby. The bankside vegetation is scrub of quila (*Chusquea quila* Kunth) and maqui (*Aristotelia chilensis* Molina) Stuntz. In six sweeps, we collected two specimens of *A. expansa*. We also collected three specimens of *A. concepcionensis* Schmitt, 1942 and seven *Aegla aff. denticulata*.
8. Chillancito Stream (50 m a.s.l.) is a permanent stream approximately 1 m wide with very dense submerged and floating aquatic vegetation. In nine sweeps, we captured one specimen of *A. expansa*. The stream runs through fields planted with subsistence crops, with some cattle farming and is set in a matrix of forestry plantations of exotic species. It is in a surprisingly good environmental state to judge by the fish species found (e.g., *Percilia irwini* Eigenmann).
9. Quilacoya River at Vegas de Diuca (2 m a.s.l.). The river has a very broad, flat, sand-and-quartzite-mica gravel bottom, covered by shallow water that reaches 1 m in depth in the pools. Close to the bridge (under reconstruction) there is a patch of gravel, the only place where any specimens were collected. It is surrounded by arboreal vegetation and mixed scrub, predominantly of exotic species. In nine sweeps we collected 16 specimens of *A. expansa*. The local area is highly disturbed by construction work and is set within a matrix of exotic-forest plantations.
10. Quilacoya River at a bridge close to its confluence with the Biobío River (30 m a.s.l.). The bottom is sand and quartziferous and mica gravel, with a patch of round stones and rubble below the bridge where the track crosses; the water is semi-turbid, yellowish, with exotic tree species growing on the banks. In eight sweeps, no specimens of *A. expansa* were collected, but eight specimens of *Aegla* sp. (aff. araucaniensis) were recovered. Rubbish was found on the banks.
11. Biobío River beside the main road, near the village of Quilacoya, between the two railway crossings (34 m a.s.l.). In this area the river is sandy, with artificial clasts on the banks. The bottom consists of mud and gravel flooded by the rise in the river after heavy rains during the collection season. The water was semi-turbid. In five sweeps, no specimens of *Aegla* spp. were recovered.

### Endemism and distribution

According to Jara (2005), seven species of the genus *Aegla* are distributed in the area of the Coastal Range between the mouth of the Biobío River (Biobío region) and the mouth of the Bueno River (Los Ríos region) (*A. concepcionensis*, *A. expansa*, *A. bahamondei* Jara, 1982, *A. cholchol*, *A. spectabilis* Jara, 1986, *A. manni* Jara, 1980, and *A. hueicollensis* Jara & Palacios, 1999). They are all endemic to Chile. Jara et al. (2006) consider *A. concepcionensis* and *A. expansa* to be highly endemic species, probably restricted to one or a few interconnected hydrographic basins. This appears to be the case for *A. expansa*, but *A. concepcionensis* has been described in other basins of southern Chile, up to 270 km south of the range described previously (Correa-Araneda et al. 2022). In the present study, and in studies by other authors (e.g., Pérez-Losada et al. 2004; Valdovinos 2019; Catchpole et al. 2023), rivers and streams in neighbouring basins and micro-basins were sampled, and no specimens of *A. expansa* were recorded (e.g., Nonguén Stream, Pineda Lake, Cárcamo Stream in Concepción, Chaimávida Stream, Andalién River, stream at the NW exit from Rere, Pachagua River in Rere, Claro River in Yumbel). The greatest abundances of *A. concepcionensis* were recorded in stations 3, 4, and 9, with mean densities of 0.17 and 0.28 ind./0.1 m^2^.

### Threats and conservation proposals

Pérez-Losada et al. (2002) proposed that *A. expansa* and *A. concepcionensis* were Extinct in the Wild (EW) “because exhaustive studies over several years in known and expected habitats across their historical ranges have not recorded a single individual. The presence of both species has been severely altered by forestry operations and urbanization” (Jara 1992, 1996). However, we recorded *A. expansa* in nine water bodies and *A. concepcionensis* in four, all in the commune of Hualqui and within the Biobío River basin. These results and those of Correa-Araneda et al. (2022) for *A. concepcionensis*, refute the category of Extinct in the Wild (EW) for both species. Furthermore, in three water bodies we recorded sympatric populations of *A. expansa* and *A. concepcionensis* (La Araucana Stream, El Relajo Stream, and Quilacoya River).

There is an urgent need for a conservation strategy for the genus *Aegla*, as has been proposed by various authors (e.g., Pérez-Losada et al. 2002; Jara et al. 2006; Santos et al. 2017). It is particularly urgent in the case of *Aegla expansa* as an endangered microendemic species; the strategy should focus on (a) habitat restoration and (b) study of its natural history.

Habitat conservation requires consideration of current land use in the commune of Hualqui, which is dominated by forestry plantations of *Pinus radiata* (D. Don) and *Eucalyptus* spp. The cycle of planting, harvesting, and replanting these species, which has been ongoing in Hualqui for decades, results in habitat destruction because stream beds become clogged with sand and sediment washed down from adjacent hillsides. The first step would be to enforce current legislation (e.g., Supreme Decree N° 2.374, Law 20.283, Law 20.283), which requires that native vegetation be maintained along the banks of water bodies. This is not complied with at any of the sites. For more details of laws and their application, see Möller and Muñoz-Pedreros (2014). Recovery of this bankside vegetation will improve the quantity and quality of water in rivers and streams and regulate temperature and light in aquatic systems (Scarsbrook et al. 2001; Sirombra and Mesa 2010; Romero et al. 2014). The basic principles of ecological restoration (*sensu*SER 2004) are well known, and experiments have been carried out in Chile (Smith-Ramírez et al. 2015; Muñoz-Pedreros et al. 2021); we therefore propose a recovery plan for banksides by replanting with species native to the area.

We also found severe alteration of the habitat due to the accumulation of non-degradable domestic rubbish in stream beds and along the banks, especially in the proximity of bridges and fords (e.g., drink cans, disposable nappies, clothing, mattresses, polyethylene sacks, sanitary towels, plastic supermarket bags, shoes, building rubble, furniture). Extraction and control of this rubbish should be coordinated by the municipality of Hualqui.

Learning about the natural history of this species is fundamental (e.g., habitat requirements, trophic ecology, reproduction). A chromosome number of 2n = 154 was found in somatic cells of specimens of *A. expansa* obtained from the San Onofre Stream in the present study. This chromosome number is high considering their small size (< 2 μm) (Jara-Seguel et al. 2020). These cytogenetic data are the first reported for a species of the genus *Aegla*; they therefore contribute knowledge of a basic genome characteristic, which in the future could be applied to understanding other biological features of these freshwater crustaceans (Jara-Seguel et al. 2020). The molecular phylogenetic position of *A. expansa* suggests a near relationship with *A. araucaniensis*, a species with a wide distribution in south-central Chile but not present in the same geographic area as *A. expansa*. However, further surveys of the area could confirm the presence of other *Aegla* species in this zone. The freshwater decapod crustacean collection of the CEA holds specimens from the river wetlands described previously, which we hope can be used as basic material for investigation in the immediate future.

## Additional information

### Use of AI

No use of AI was reported.

### Author ORCIDs

Andrés Muñoz-Pedreros https://orcid.org/0000-0001-6363-6549

Heraldo V. Norambuena https://orcid.org/0000-0003-0523-3682

Carlos G. Jara https://orcid.org/0000-0001-5337-7985

Pedro Jara-Seguel https://orcid.org/0000-0002-9340-1780

### Data availability

All of the data that support the findings of this study are available in the main text or Supplementary Information.

## Supplementary materials

Supplementary material 1 Sequences deposited in GenBank Data type docx Explanation note Taxon sample list, including institution, locality, and GenBank accession number. https://binary.pensoft.net/file/1527391This dataset is made available under the Open Database License (http://opendatacommons.org/licenses/odbl/1.0/). The Open Database License (ODbL) is a license agreement intended to allow users to freely share, modify, and use this Dataset while maintaining this same freedom for others, provided that the original source and author(s) are credited. Andrés Muñoz-Pedreros, Heraldo V. Norambuena, Carlos G. Jara, Pedro Jara-Seguel, Marcela Guerrero, Marilyn González-Urrutia
