# Supplementary material for: The microendemic Aegla expansa (Aeglidae) survives in highly disturbed micro-basins of southern Chile
Source: Zookeys. 2026 Feb 4;1268:61–73. doi: 10.3897/zookeys.1268.167269 (PMC12895179; doi:10.3897/zookeys.1268.167269)
Supplement: Supplementary material 1 — Sequences deposited in GenBank [file zookeys-1268-061_article-167269__-s001.docx]

Table S1. Taxon sample list, including institution, locality, and genBank accession number per locus.

| N° | Taxon | Locality | COI | Reference |
| --- | --- | --- | --- | --- |
| 1 | *Aegla concepcionensis* | - | FJ360704 | Pérez-Losada et al. 2009 |
| 2 | *Aegla concepcionensis* | - | FJ360705 | Pérez-Losada et al. 2009 |
| 3 | *Aegla cholchol* | Imperial River, Chol-Chol River, Chile | HQ236296 | Bracken-Grissom et al. 2011 |
| 4 | *Aegla cholchol* | Imperial River, Chol-Chol River, Chile | HQ236295 | Bracken-Grissom et al. 2011 |
| 5 | *Aegla rostrata* | Valdivia River, Riñihue Lake, Chile | HQ236257 | Bracken-Grissom et al. 2011 |
| 6 | *Aegla rostrata* | Valdivia River, Riñihue Lake, Chile | HQ236256 | Bracken-Grissom et al. 2011 |
| 7 | *Aegla laevis talcahuano* | Lircay River, Maule River, Chile | AY050087 | Pérez-Losada et al. 2002 |
| 8 | *Aegla laevis talcahuano* | Torreón River, Ñuble River, Chile | AY050086 | Pérez-Losada et al. 2002 |
| 9 | *Aegla laevis laevis* | Trebulco Creek, Maipo River, Chile | AY050084 | Pérez-Losada et al. 2002 |
| 10 | *Aegla laevis laevis* | Trebulco Creek, Maipo River, Chile | AY050083 | Pérez-Losada et al. 2002 |
| 11 | *Aegla pewenchae* | Bío Bío River, Icalma Lake, Chile | AY050101 | Pérez-Losada et al. 2002 |
| 12 | *Aegla pewenchae* | Bío Bío River, Icalma Lake, Chile | AY050100 | Pérez-Losada et al. 2002 |
| 13 | *Aegla araucaniensis* | Chaimávida Creek, Bío Bío River, Chile | AY050089 | Pérez-Losada et al. 2002 |
| 14 | *Aegla araucaniensis* | Chaimávida Creek, Bío Bío River, Chile | AY050088 | Pérez-Losada et al. 2002 |
| 15 | *Aegla spectabilis* | Imperial River, Chol-Chol River, Chile | AY050097 | Pérez-Losada et al. 2002 |
| 16 | *Aegla abtao* | Valdivia River, Riñihue Lake, Chile | AY050109 | Pérez-Losada et al. 2002 |
| 17 | *Aegla abtao* | Bueno River, Rupanco Lake, Chile | AY050108 | Pérez-Losada et al. 2002 |
| 18 | *Aegla riolimayana* | Negro River, Moquehue-Aluminé Lake, Argentina | AY595548 | Pérez-Losada et al. 2002 |
| 19 | *Aegla riolimayana* | Negro River, Moquehue-Aluminé Lake, Argentina | AY050098 | Pérez-Losada et al. 2002 |
| 20 | *Aegla affinis* | Maule River, Chile | AY050082 | Pérez-Losada et al. 2002 |
| 21 | *Aegla affinis* | Maule River, Chile | AY050081 | Pérez-Losada et al. 2002 |
| 22 | *Aegla bahamondei* | Huillinco Creek, Tucapel River, Chile | AY050090 | Pérez-Losada et al. 2002 |
| 23 | *Aegla alacalufi* | Rosselot Lake, Chile | FJ471741 | Xu et al. 2009 |
| 24 | *Aegla manni* | Valdivia River, Chile | AY050118 | Pérez-Losada et al. 2002 |
| 25 | *Aegla manni* | Valdivia River, Chile | AY050117 | Pérez-Losada et al. 2002 |
| 26 | *Aegla denticulata lacustris* | Bueno River, Rupanco Lake, Chile | AY050115 | Pérez-Losada et al. 2002 |
| 27 | *Aegla denticulata lacustris* | Bueno River, Rupanco Lake, Chile | AY050114 | Pérez-Losada et al. 2002 |
| 28 | *Aegla denticulata denticulata* | Bueno River, Chifín River, Chile | AY050107 | Pérez-Losada et al. 2002 |
| 29 | *Aegla denticulata denticulata* | Bueno River, Chifín River, Chile | AY050106 | Pérez-Losada et al. 2002 |
| 30 | *Aegla hueicollensis* | Bueno River, Hueicolla River, Chile | AY050103 | Pérez-Losada et al. 2002 |
| 31 | *Aegla hueicollensis* | Bueno River, Hueicolla River, Chile | AY050102 | Pérez-Losada et al. 2002 |
| 32 | *Aegla papudo* | Aconcagua River, Rabuco River, Chile | AY050078 | Pérez-Losada et al. 2002 |
| 33 | *Aegla expansa* | La Araucana stream, Hualqui, Chile | Pending | This study |
| 34 | *Aegla expansa* | San Onofre stream, Hualqui, Chile | Pending | This study |
| 35 | *Aegla lancinhas* | - | FJ360707 | Pérez-Losada et al. 2009 |
| 36 | *Aegla logirostri* | Taquari-Guaíba River, Brazil | AY595610 | Pérez-Losada et al. 2004 |
| 37 | *Aegla ludwigi* | Brazil | MH212140 | Zimmermann et al. 2019 |
| 38 | *Aegla franca* | Brazil | MH998637 | Bartholomei-Santos et al. 2019 |
| 39 | *Aegla carinata* | Brazil | MH998636 | Bartholomei-Santos et al. 2019 |
| 40 | *Aegla brevipalma* | Brazil | MH998635 | Bartholomei-Santos et al. 2019 |
| 41 | *Aegla georginae* | Brazil | MG581892 | Zimmermann et al. 2019 |
| 42 | *Aegla platensis* | Guaíba River, Brazil | MG581880 | Zimmermann et al. 2019 |
| 43 | *Aegla spinipalma* | Ivaí River, Jacuí-Guaíba River, Brazil | KX910293 | Pérez-Losada et al. 2004 |
| 44 | *Aegla neuquensis* | Collón Curá River, Negro River, Argentina | AY595668 | Pérez-Losada et al. 2004 |
| 45 | *Aegla intercalata* | Las Carreras Creek, Dulce River, Argentina | AY595666 | Pérez-Losada et al. 2004 |
| 46 | *Aegla humahuaca* | Sali River, Dulce River, Argentina | AY595661 | Pérez-Losada et al. 2004 |
| 47 | *Aegla ringueleti* | Calchaqui River, Salado River, Argentina | AY595657 | Pérez-Losada et al. 2004 |
| 48 | *Aegla sanlorenzo* | Los Berros Creek, Bermejo River, Argentina | AY595655 | Pérez-Losada et al. 2004 |
| 49 | *Aegla jujuyana* | Grande River, Bermejo River, Argentina | AY595653 | Pérez-Losada et al. 2004 |
| 50 | *Aegla septentrionalis* | Yavi Creek, Pilcomayo River, Argentina | AY595651 | Pérez-Losada et al. 2004 |
| 51 | *Aegla scamosa* | Desaguadero River, Argentina | AY595649 | Pérez-Losada et al. 2004 |
| 52 | *Aegla obstipa* | Horto Forestal Ramos Creek, Guaíba River, Brazil | AY595646 | Pérez-Losada et al. 2004 |
| 53 | *Aegla strinatii* | Ribeira do Iguape River, Brazil | AY595640 | Pérez-Losada et al. 2004 |
| 54 | *Aegla leptochela* | Ribeira do Iguape River, Brazil | AY595638 | Pérez-Losada et al. 2004 |
| 55 | *Aegla cavernicola* | Areia II Cave River, Ribeira Iguape River, Brazil | AY595636 | Pérez-Losada et al. 2004 |
| 56 | *Aegla prado* | Montevideo, La Plata River, Uruguay | AY595634 | Pérez-Losada et al. 2004 |
| 57 | *Aegla uruguayana* | del Palacio Cave, Uruguay River, Uruguay | AY595632 | Pérez-Losada et al. 2004 |
| 58 | *Aegla violacea* | Guaíba River, Brazil | AY595626 | Pérez-Losada et al. 2004 |
| 59 | *Aegla rossiana* | Mampituba-Litoral River, Brazil | AY595624 | Pérez-Losada et al. 2004 |
| 60 | *Aegla inermis* | Caará Creek, Sinos-Guaíba River, Brazil | AY595622 | Pérez-Losada et al. 2004 |
| 61 | *Aegla camargoi* | Pelotas River, Brazil | AY595620 | Pérez-Losada et al. 2004 |
| 62 | *Aegla leptodactyla* | Divisa Creek, Pelotas River, Brazil | AY595619 | Pérez-Losada et al. 2004 |
| 63 | *Aegla serrana* | Guirra Creek, Caí-Guaíba River, Brazil | AY595617 | Pérez-Losada et al. 2004 |
| 64 | *Aegla grisella* | Taquari-Guaíba River, Brazil | AY595616 | Pérez-Losada et al. 2004 |
| 65 | *Aegla spinipalma* | Jacuí-Guaíba River, Brazil | AY595615 | Pérez-Losada et al. 2004 |
| 66 | *Aegla singularis* | Ijuí-Pelotas River, Brazil | AY595614 | Pérez-Losada et al. 2004 |
| 67 | *Aegla franciscana* | Guaíba River, Brazil | AY595606 | Pérez-Losada et al. 2004 |
| 68 | *Aegla inconspicua* | Guaíba River, Brazil | AY595604 | Pérez-Losada et al. 2004 |
